# Health and Aging Disparities Among Latin American Older Adults: Findings From Studies in Brazil, Colombia, and Mexico

**DOI:** 10.1093/geroni/igab046.2272

**Published:** 2021-12-17

**Authors:** Margarita Osuna

## Abstract

Aging in Latin America is occurring rapidly, in a context of high levels of poverty and inequality. This symposium is focused on population health and the heath-disparities found in some of Latin America’s largest middle-income countries, Mexico, Brazil, and Colombia. This symposium contains presentations on different health-related issues affecting older adults, which can have further implications for wellbeing, health, and disease risk. The papers in this symposium examine a variety of health-related dimensions and disparities among older Latinos that include physiological functioning, cognition, and oral health. Using the Mexican Health and Aging Study (MHAS), Sheehan investigates the associations between personal and familial educational attainment on sleep quality. Also using MHAS, Milani. Using data from the Brazilian Longitudinal Study of Aging, Farina examines the relationship between race and cognition. Garcia uses data from the Colombian Survey of Health, Well-Being, and Aging to study the relationship between Motor Cognitive Risk Syndrome (MCRS) and cognition and frailty. Using the same dataset, Osuna examines variation in oral health in Colombian older adults and the impact this has on their wellbeing. Results indicate which population subgroups in Latin American have increased risk for poorer health and which dimensions of health have gender, race and socioeconomic disparities. The findings highlight the importance of understanding the conditions under which Latin American older adults are aging and the implications this can have in the future.

